# The Transcription Factor Hand1 Is Involved In Runx2-Ihh-Regulated Endochondral Ossification

**DOI:** 10.1371/journal.pone.0150263

**Published:** 2016-02-26

**Authors:** Lindsay E. Laurie, Hiroki Kokubo, Masataka Nakamura, Yumiko Saga, Noriko Funato

**Affiliations:** 1 Research Center for Medical and Dental Sciences, Tokyo Medical and Dental University (TMDU), Bunkyo-ku, Tokyo, Japan; 2 Division of Mammalian Development, National Institute of Genetics, Mishima, Shizuoka, Japan; 3 Department of Genetics, The Graduate University for Advanced Studies, Mishima, Shizuoka, Japan; 4 Department of Cardiovascular Physiology and Medicine, Graduate School of Biomedical and Health Sciences, Hiroshima University, Minamiku, Hiroshima, Japan; Kyungpook National University School of Medicine, REPUBLIC OF KOREA

## Abstract

The developing long bone is a model of endochondral ossification that displays the morphological layers of chondrocytes toward the ossification center of the diaphysis. Indian hedgehog (Ihh), a member of the hedgehog family of secreted molecules, regulates chondrocyte proliferation and differentiation, as well as osteoblast differentiation, through the process of endochondral ossification. Here, we report that the basic helix-loop-helix transcription factor Hand1, which is expressed in the cartilage primordia, is involved in proper osteogenesis of the bone collar via its control of *Ihh* production. Genetic overexpression of *Hand1* in the osteochondral progenitors resulted in prenatal hypoplastic or aplastic ossification in the diaphyses, mimicking an *Ihh* loss-of-function phenotype. *Ihh* expression was downregulated in femur epiphyses of *Hand1-*overexpressing mice. We also confirmed that Hand1 downregulated *Ihh* gene expression *in vitro* by inhibiting Runx2 transactivation of the *Ihh* proximal promoter. These results demonstrate that Hand1 in chondrocytes regulates endochondral ossification, at least in part through the Runx2-Ihh axis.

## Introduction

The vertebrate skeleton develops through two tightly controlled processes: intramembranous ossification and endochondral ossification. Intramembranous ossification is the direct differentiation of condensed mesenchymal cells into the osteoblasts of bone tissue. Endochondral ossification is characterized by condensation of mesenchymal cells to produce a cartilage primordium surrounded by the perichondrium, consisting of prechondroblasts, osteoblasts, and fibroblasts [1,2]. The long bones, developed by endochondral ossification, consist of two cartilaginous epiphyses connected by a bony diaphysis. The process of ossification begins in two locations; the primary ossification is located in the center of the future diaphysis and the secondary ossification is located in the center of the epiphysis.

Indian hedgehog (Ihh) is a member of the hedgehog family of secreted molecules, which controls chondrocyte proliferation and differentiation as well as osteoblast differentiation. *Ihh* is detected in the chondrocytes of the early cartilage primordium [3]. *Ihh*^-/-^ mice display severely shortened long bones, fused digits, delayed calcification, and a failure of cortical bone and bone collar formation [4]. Ectopic expression of *Ihh* in chondrocytes induces expression of Runx2 (Runt-related transcription factor 2), a master molecule for osteoblast differentiation, throughout the perichondrium that induces bone collar formation [5]. Temporary attenuation of Ihh activity decreased Runx2 expression and produced mice with shortened limbs, trunk and skull bones [6]. Deletion of *Runx2* disables the expression of *Ihh*; however, the addition of *Runx*2 restores *Ihh* expression [2]. Thus, Runx2 positively regulates *Ihh* expression in chondrocytes, and, in turn, *Ihh* also positively regulates *Runx2* expression in the perichondrium; disruption of the latter process results in impaired chondrocyte differentiation and osteoblastogenesis.

Basic helix-loop-helix (bHLH) transcription factors played the crucial roles during embryonic development. Hand1 and Hand2, highly conserved bHLH proteins, are expressed in the developing limb bud [7,8,9]. Genomic regions enriched in Hand2 chromatin complexes were identified in early limb buds [10]. In *Hand2* transgenic mice, bones of the zeugopod, in both forelimbs and hindlimbs, were shortened and malformed [8]. However, little is known about the role of Hand1 and Hand2 in the development of the endochondral bones. Here, we demonstrate that *Hand1-*overexpressing mice show aplastic or hypoplastic ossification in the long bones, partially mimicking the bone phenotype observed in *Ihh*^*-/-*^ mice. Hand1 inhibits *Ihh* expression by suppressing Runx2 transactivation of the *Ihh* promoter. Our data indicate that Hand1 acts as a negative regulator of endochondral ossification.

## Materials and Methods

### *Hand1* conditionally-overexpressing mice

The transgene vector *CAG*-lox-*CAT*-lox-*Hand1* was constructed by inserting a *Hand1* cDNA into the *CAG-CAT-*(cDNA insert)-poly(A) cassette to generate a transgenic line, *CAG-CAT Hand1*^*Tg/+*^ (Stock No. RBRC01369, RIKEN). For conditional activation of *Hand1*, *Twist2-Cre* knock-in males [11] were crossed with *CAG-CAT Hand1*^*Tg/+*^ females. *Rosa26* Reporter (*R26R*) (Stock No. 6148, The Jackson Laboratory) mice have been described previously [12].

This study was carried out in strict accordance with the recommendations in the Guide for the Care and Use of Laboratory Animals of the National Institutes of Health. All animal experimental procedures were reviewed and approved by the Institutional Animal Care and Use Committee (IACUC) of Tokyo Medical and Dental University (Permit Number: 0160215A). All efforts were taken to minimize pain experienced by the mice. Animals were housed with no more than 4 per cage. All animals were maintained in an HEPA (high-efficiency particulate arrestance)-filtered rack with a 12-hour light/dark cycle. Animals were fed an autoclaved laboratory rodent diet. Animals were sacrificed by carbon dioxide inhalation.

### Bone staining

Skeletal preparations were stained using alcian blue for cartilage and alizarin red for ossified bones, as described previously [13].

### Histology and immunohistochemistry

Bone samples were fixed in 4% paraformaldehyde, decalcified, and embedded in paraffin, as previously described [14]. To unmask antigens, tissue sections were boiled in 10 mM citrate buffer [for phospho-Histone H3, and Spp1 (Secreted phosphoprotein 1)], or incubated in 700 U/mL proteinase K solution (Nacalai Tesque) (for Sox9) at 37°C for 5 minutes, or treated with 1mg/mL hyaluronidase (Sigma-Aldrich) at 37°C (for Runx2). Immunohistochemistry was performed using the Vectastain Elite ABC kit (Vector) and Immpact DAB peroxidase substrate (Vector). Sections were counterstained with Hematoxylin QS (Vector) or Methyl Green Nuclear Counterstain (Vector). The following primary antibodies were used: anti-Spp1 antibody (RB9097-PO; Thermo Scientific), anti-Ihh antibody (sc-1196; Santa Cruz), anti-RUNX2/CBFA1 antibody (PA1224; Boster Biological), anti-HAND1 antibody (GTX11846; GeneTex), anti-SOX9 antibody (AB5535; Millipore), and anti-phospho-Histone H3 (Ser10) antibody (06–570; Millipore).

For alcian blue staining, sections were treated with 3% acetic acid, stained with 1.5 mg/mL alcian blue 8GX in 3% acetic acid solution, and then counterstained with Eosin Y (Sigma-Aldrich). For von Kossa staining, sections were incubated in 1% silver nitrate overnight under UV light, then incubated in 5% sodium thiosulfate, and counterstained with Eosin Y.

### Real-time quantitative PCR (qPCR)

Total RNA was extracted from tissue or cells using TRIzol reagent (Invitrogen) according to the manufacturer’s instructions. cDNA was synthesized using 1st strand cDNA synthesis kit for RT-PCR (AMV) (Roche). Real-time quantitative PCR (qPCR) was performed using the LightCycler FastStart DNA MasterPLUS SYBR Green 1 kit (Roche). Amplification of single products was confirmed by monitoring dissociation curves. All data were normalized to *Ppia* (peptidylprolyl isomerase A) expression.

Primer sequences used for amplification were as follows: *Hand1* forward: 5′-CTTTAATCCTCTTCTCGCCG-3′, *Hand1* reverse: 5′-CAAGGATGCACAAGCAGGT-3′; *Ppia* forward: 5′- CGCGTCTCCTTCGAGCTGTTTG -3′, *Ppia* reverse: 5′- TGTAAAGTCACCACCCTGGCACAT-3′; *Runx2* forward: 5′-GCTCACGTCGCTCATCTTG-3′, *Runx2* reverse: 5′-TATGGCGTCAAACAGCCTCT-3′; and *Ihh* forward: 5′-TGACAGAGATGGCCAGTGAG-3′, *Ihh* reverse: 5′-AGAGCTCACCCCCAACTACA-3′.

### Luciferase assay and stable cell lines

ATDC5 (RIKEN) and COS1 (RIKEN) cells were grown in Dulbecco’s Minimal Essential Medium (DMEM)/Ham’s F12 or DMEM high glucose media respectively, supplemented with 10% fetal bovine serum. Luciferase assays were performed as previously described [15]. pIhh-luc [2], expression vectors for Hand1, Hand2, Tcf15 [16], Runx2 [17], Id1 [18] and Tcf21 [19] were described previously.

ATDC5 cells were transfected with myc-Hand1 or myc-pcDNA3.1 using FuGENE 6 (Roche). For stable transfections, cells were selected using 200 μg/ml neomycin (Sigma-Aldrich) and individual clones were amplified prior to analysis. Immunostaining and qPCR verified the presence of the transgene.

### Statistical analysis

All experiments were performed independently with a minimum of three replicates. Data were analyzed using an unpaired Student’s *t*-test and expressed as the mean ± standard deviation (SD). *P*-values less than 0.05 were considered significant for all experiments; asterisks denote significance.

## Results and Discussion

### Overexpression of *Hand1* causes developmental defects in the limbs

**Fig 1:**
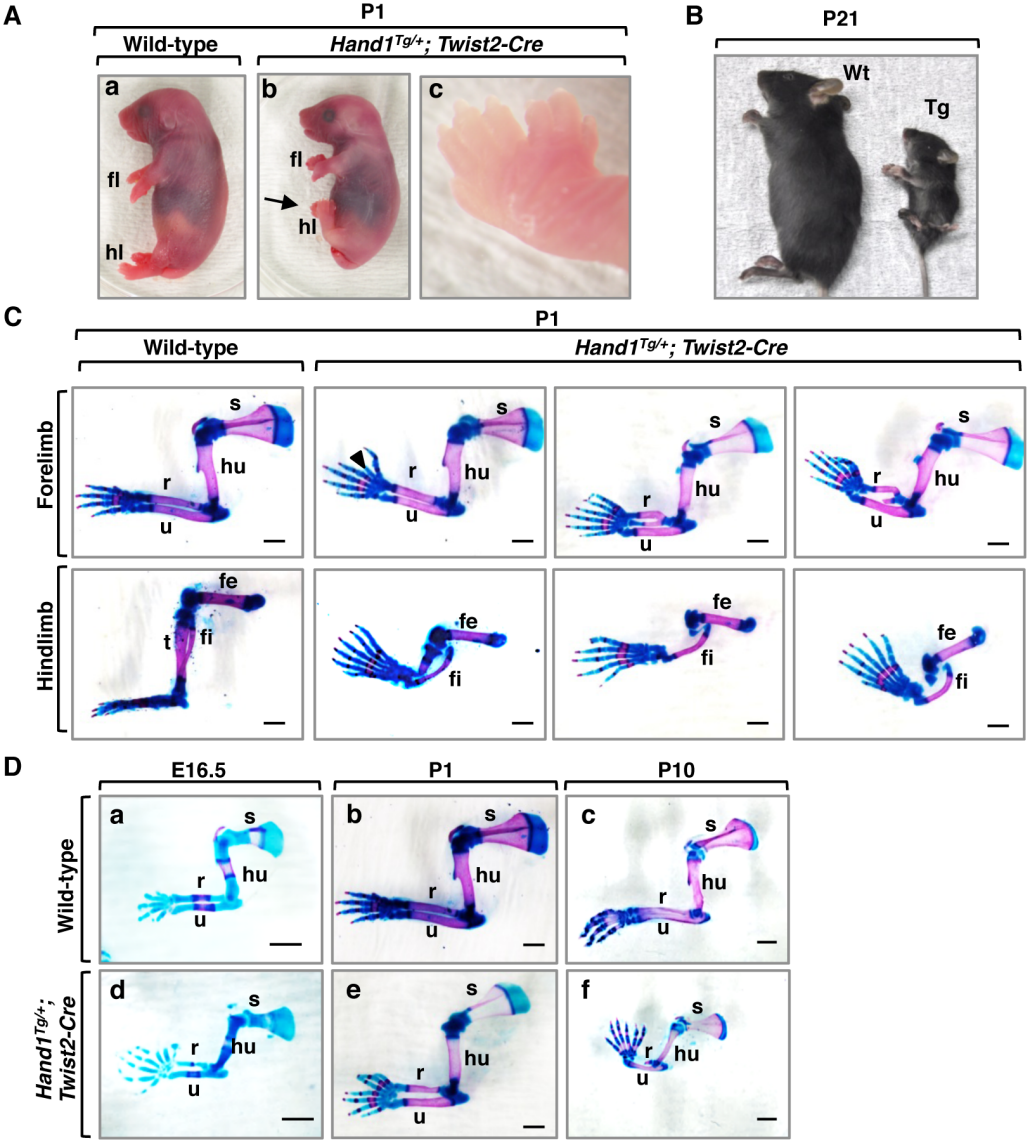
Defective patterning and ossification in *Hand1* mutant long bones. (A) Wild-type (a) and *Hand1*^*Tg/+*^*; Twist2-Cre* mutants (b,c) at P1. (B) *Hand1*^*Tg/+*^*; Twist2-Cre* mutant (Tg) and wild-type (Wt) littermate at P21. (C) Bone staining of P1 Wt and *Hand1* mutant forelimbs (top panels) and hindlimbs (bottom panels), as indicated. Scale Bars: 1mm. (D) Bone staining of Wt and *Hand1* mutants at E16.5, P1, and P10, as indicated. Scale Bars: 1 mm (a,b,d,e), 2 mm (c,f). fl, forelimb; hl, hindlimb; r, radius; u, ulna; hu, humerus; s, scapula; fe, femur; t, tibia; fi, fibula.

To investigate the role of Hand1 in the development of the endochondral bones, conditional *Hand1* transgenic mice (*Hand1*^*Tg/+*^*; Twist2-Cre*), whose *Hand1* overexpression is driven by the *Twist2* promoter in the osteochondral progenitors, were generated. During endochondral ossification, *Twist2* promoter-driven *Cre* expression is detected in the chondrocytes of the growth plate cartilage and the osteoblasts in the perichondrium, periosteum, and endosteum [11]. *Hand1*^*Tg/+*^*; Twist2-Cre* mutants were slightly dwarfed at postnatal day 1 (P1) (Fig 1A and S1 Fig). All *Hand1* mutants displayed preaxial polydactyly in the autopod (Fig 1A and S1 Table, n = 54). By P21, *Hand1* mutants were severely dwarfed (Fig 1B), and only 33% (n = 18/54) grew to adulthood. Bone staining showed hypoplastic ossification of the zeugopod; malformed, duplicated or malarticulated radii; and mirror-image duplication of digits in *Hand1* mutant forelimbs (Fig 1C and S1 Table). In *Hand1* mutant hindlimbs, aplastic ossification of tibiae, “C”-shaped fibulae, and distal phalangeal duplications were noted (Fig 1C and S1 Table). In addition, incomplete fusion of the xiphoid process and the hypoplastic supraoccipital bone were observed in the endochondral bones of *Hand1* mutants (S1 Fig). A range of malformations in endochondral ossification was already present as early as E16.5 (Fig 1D and S1 Fig). These findings suggest that *Hand1* overexpression may interfere with the commitment of limb mesenchyme cells to the cartilage fate and/or control the development of endochondral ossification.

To further investigate the role of the closely related bHLH protein Hand2 in limb development, we examined the skeletal phenotype of *Hand2*^*Tg/+*^*; Twist2-Cre* mice. *Hand2* mutants (100%, n = 10) were perinatal lethal, accompanied by skeletal abnormalities similar to those seen in *Hand1* mutants (S2 Fig). Patients with 4q trisomy: dup (4)(q35.2-q31.22) manifest preaxial polydactyly [20]. The trisomic region contains *HAND2*, and overdosage of *Hand2* is a major cause of the limb phenotypes of 4q trisomy [21]. Since *Hand1*-overexpressing mice have limb and skeletal phenotypes, the *HAND1* coding region (5q33.2) may be one of the candidate regions for preaxial defects and short stature in humans.

### *Hand1* mutants show delayed and hypoplastic ossification

**Fig 2:**
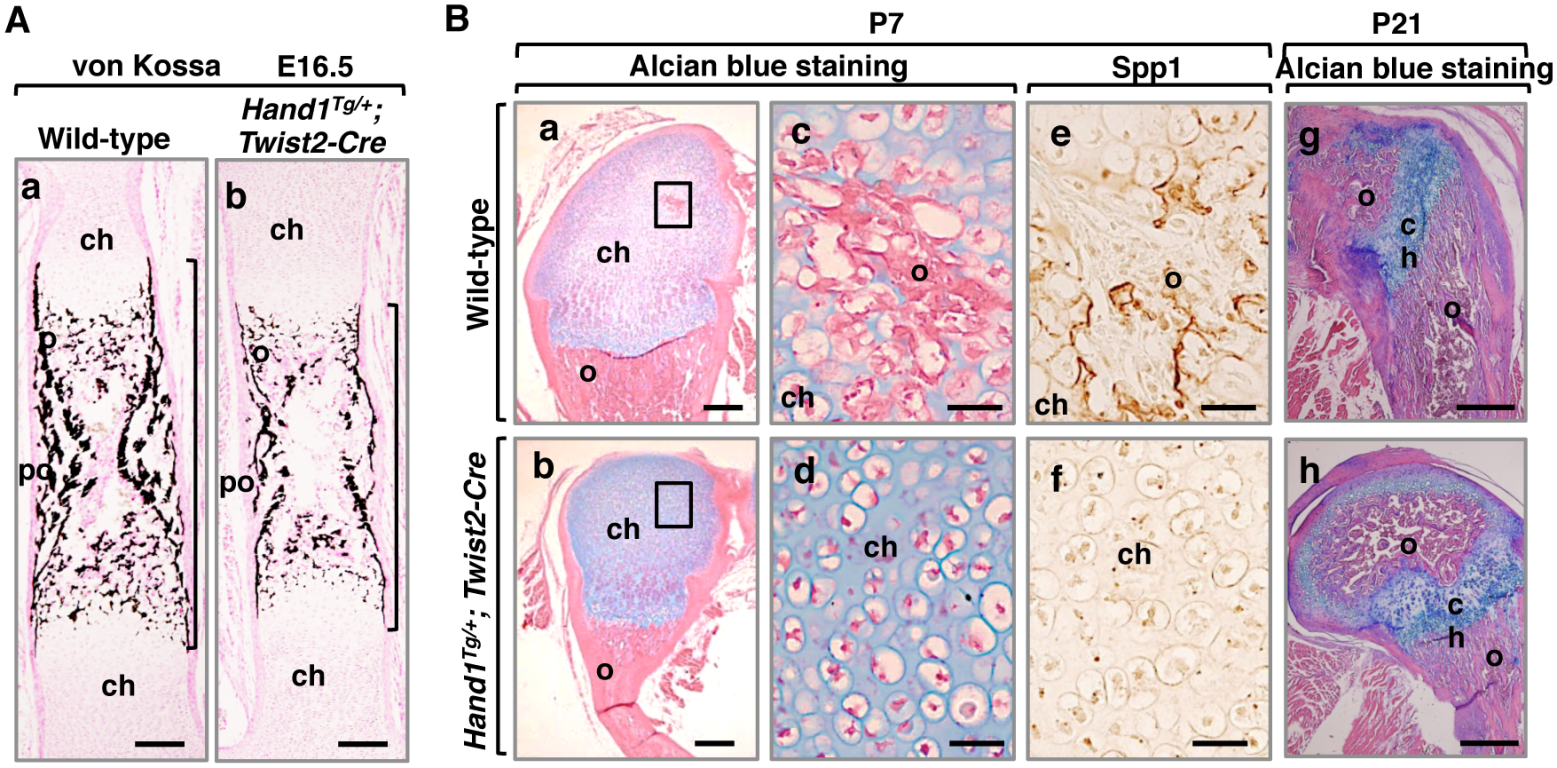
*Hand1* mutants present hypoplastic primary and secondary ossifications. (A) Hypoplastic primary ossification in *Hand1* mutant femurs. (a,b) von Kossa staining of Wt (a) and *Hand1* mutant (b) femurs at E16.5. Brackets indicate the osoteogenesis domain. Scale bars: 200 μm. (B) Delayed secondary ossification in *Hand1* mutant femurs (b,d,f,h) compared to Wt (a,c,e,g) at P7 and P21. (c,e) and (d,f) show enlargement of the boxed regions of the adjacent panels (a) and (b), respectively. Immunohistochemistry for Spp1 (e,f) indicates the presence of osteoblasts. ch, chondrocytes; po, periosteum; o, ossified tissue. Scale bars: 200 μm (a,b), 50 μm (c-f), 400 μm (g,h).

Since malformation of long bones was observed in *Hand1* and *Hand2* mutants, we focused our attention on these bone elements. von Kossa staining revealed hypoplastic bone collars in the trabeculae of *Hand1* mutant femurs at E16.5 (Fig 2A), whereas the growth plate showed no significant difference from wild-type (Wt) (S3 Fig). Expression of Sox9, an essential transcription factor in chondrocyte differentiation, and the number of dividing chondrocytes in the femoral epiphysis of *Hand1* mutants was not significantly different from Wt at E16.5 (S3 Fig).

Delayed onset of secondary ossification was also observed in *Hand1* mutants (Fig 2B). At P7, secondary ossification was observed in Wt femurs, whereas only the beginning of chondrocyte hypertrophy was visible in *Hand1* mutant femurs (Fig 2B). In Wt, Spp1 (secreted phosphoprotein 1), also known as osteopontin, was expressed in mature osteoblasts in the region of the secondary ossification center, whereas Spp1 was not expressed in the cartilaginous epiphyses in *Hand1* mutants (Fig 2B). At P21, femurs of *Hand1* mutants showed the presence of secondary ossification (Fig 2B). These results suggest that *Hand1* overexpression affects primary ossification at the prenatal stage of endochondral ossification and secondary ossification may also be controlled, at least in part, by Hand1.

### Hand1, Runx2, and Ihh are expressed in the cartilage primordia

**Fig 3:**
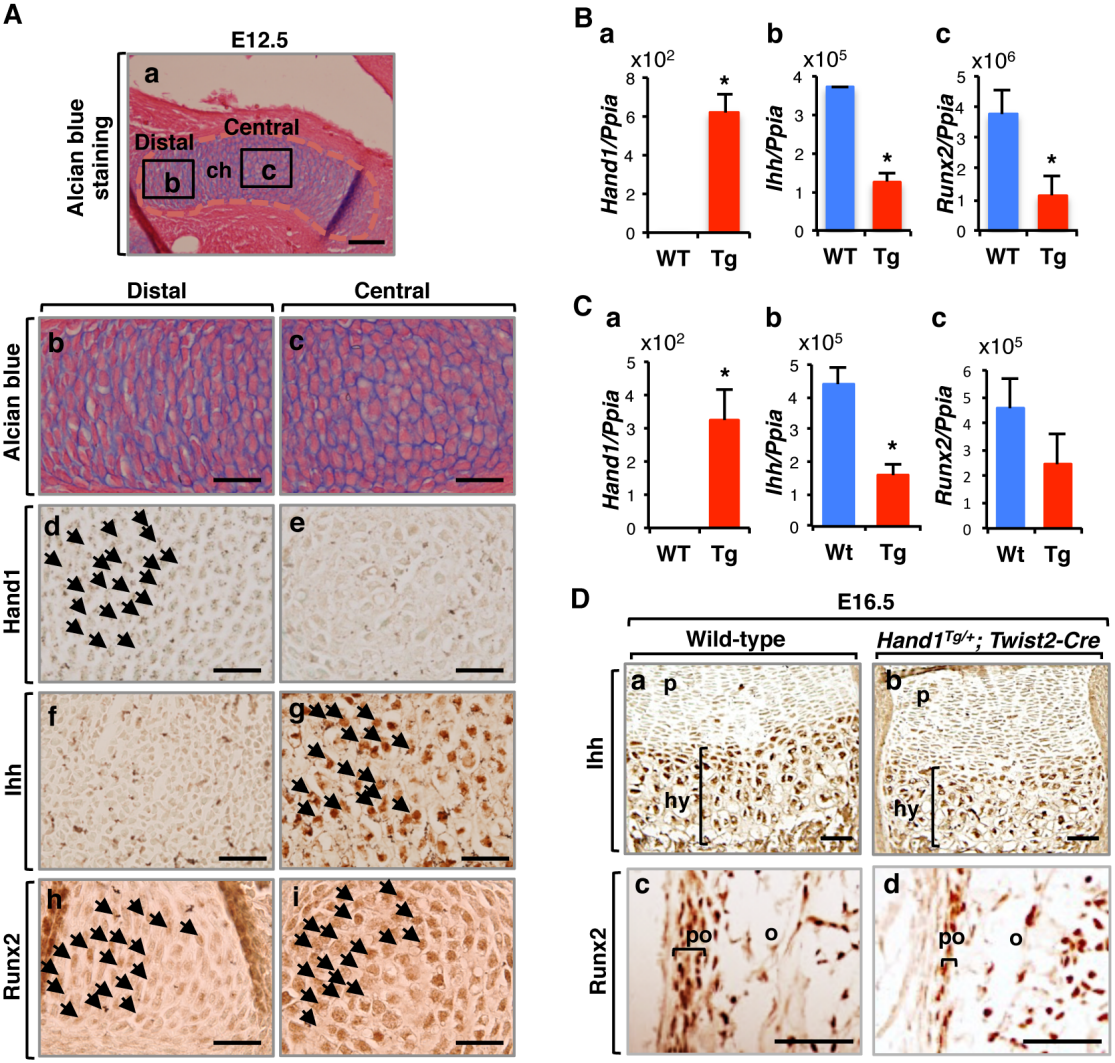
Hand1 downregulates *Ihh* and *Runx2* expression *in vivo*. (A) Endogenous expression of Hand1, Ihh and Runx2 in E12.5 cartilage primordium. Alcian blue staining (a-c) and immunohistochemical analysis for Hand1 (d,e), Ihh (f,g), and Runx2 (h,i) in the distal (b,d,f,h) and central (c,e,g,i) part of a wild-type forelimb cartilage primordium at E12.5. (b,c) Magnified images of the distal and central regions inside of the boxes in (a). Hand1 is strongly expressed in the distal part of the cartilage primordium (arrows in d). The expression pattern of Ihh (f,g) is opposite to Hand1 (d,e). Runx2 is expressed both in the distal (h) and the central part (i) of the immature chondrocytes. ch, chondrocytes. Scale bars: 100 μm (a), 25 μm (b-i). (B, C) qPCR analysis of *Hand1* (a), *Ihh* (b) and *Runx2* (c) transcripts in femoral epiphyseal cartilage at E16.5 (B) and P1 (C). All data were normalized to *Ppia* expression. (D) Expression of Ihh (a,b) and Runx2 (c,d) was detected by immunohistochemistry of E16.5 Wt (a) and *Hand1* mutant (b) femurs. The zone of Ihh-positive chondrocytes (brackets in a,b) and the region of Runx2-positive preosteoblasts in the periosteum (brackets in c,d) is decreased in *Hand1* mutants compared to Wt. p, proliferating chondrocytes; hy, hypertrophic chondrocytes; po, periosteum; o, ossified tissue. Scale bars: 50 μm.

The above results suggested that the bone abnormalities in *Hand1* mutants are induced by a primary defect in the regulation of endochondral ossification. To examine whether the *Hand1* expression pattern is compatible with such a phenotype, immunostaining was performed in the cartilage primordium of the forelimb. At E12.5, Hand1 was strongly expressed in the distal and proximal parts of immature chondrocytes (Fig 3A). At E16.5, no detectable *Hand1* signal in Wt femoral epiphyseal cartilage was demonstrated by qPCR, whereas *Hand1* was overexpressed in mutant femurs (Fig 3B). These results suggest a potential role for Hand1 at the primordial stage of cartilage development.

The phenotypic abnormalities noted in long bone ossification in *Hand1* mutants are partially reminiscent of those observed in *Ihh*^-/-^ mice [4] and *Runx2*^+/-^ mice [22], which exhibit delayed primary ossification and failure of bone collar formation. Given the expression of Hand1 in the cartilage primordia and the defective ossification seen in *Hand1* mutants, we examined the expression of Runx2 and Ihh in the cartilage primordium of the forelimb at E12.5. In contrast to Hand1 expression, Ihh was mainly expressed in the central part of the cartilage primordium (Fig 3A). Runx2 was expressed in the whole cartilage template, although more strongly in the centrally located chondrocytes (Fig 3A). Taken together, these results suggest that Runx2 is coexpressed with Ihh and Hand1 in the cartilage primordium, while the expression pattern of Ihh is opposite to that of Hand1.

### *Ihh* expression is decreased in the epiphyseal cartilage of *Hand1* mutants

To further analyze whether Hand1 genetically regulates the expression of *Ihh* contributing to endochondral ossification, we examined gene expression by qPCR of femoral epiphyseal cartilage. Expression of *Ihh* and *Runx2* was significantly decreased in *Hand1* mutant epiphyseal cartilage at E16.5 and P1 (Fig 3B and 3C). We also confirmed by immunohistochemistry that the *Ihh*-positive region was decreased in *Hand1* mutant hypertrophic chondrocytes at E16.5 (Fig 3D). During endochondral ossification, osteoprogenitor cells in the perichondrium give rise to osteoblasts. Osteoblasts then enter the cavity via periosteal buds and deposit osteoid on the calcified matrix as a scaffold [1]. The immunohistochemistry analysis of Runx2 showed reduction in the number of Runx2-positive osteoblasts in the periosteum and the perichondrium of *Hand1* mutant femurs (Fig 3D). *Hand1* mutant femurs showed more fibrous periosteum and less cellular periosteum (Fig 3D). These results indicate that Hand1 affects *Ihh* and *Runx2* expression in the early development of the cartilage. It is also possible that Hand1 is involved in the regulation of cell population expressing *Runx2* in the periosteum.

### Hand1 negatively regulates *Ihh* expression *in vitro*

**Fig 4:**
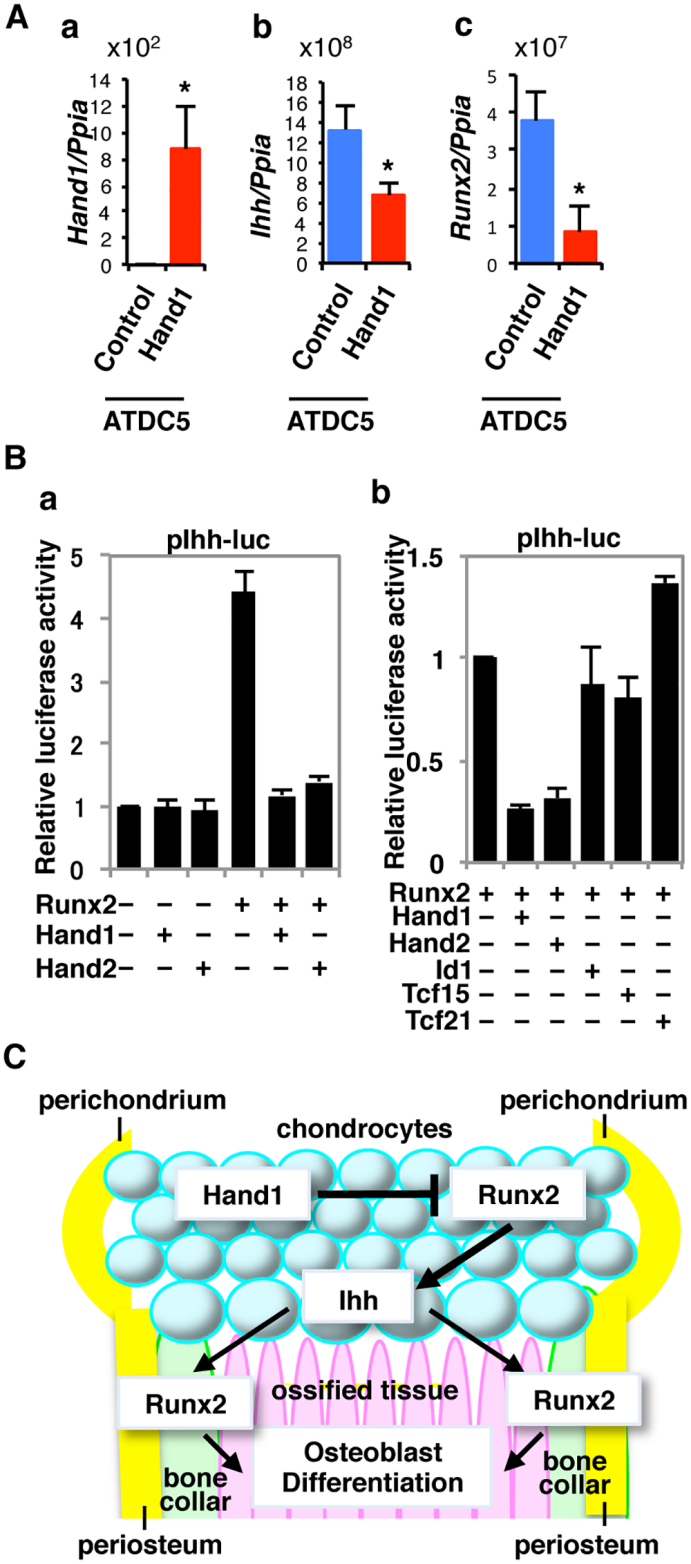
Hand1 inhibits the *Ihh* promoter through Runx2 transactivation. (A) qPCR analysis of *Hand1* (a), *Ihh* (b), and *Runx2* (c) transcripts in ATDC5 cells stably transfected with an empty vector (Control) or *Hand1* expression vector. (B) Luciferase assays. COS1 cells were transiently cotransfected with pIhh-luc reporter and the indicated expression vectors. Luciferase data (b) is shown as a percentage of Runx2 activation (normalized to 1.0). The data represent the mean ± SD. (C) Model for transcriptional regulation of endochondral ossification by Hand1. Hand1 inhibits Runx2-dependent *Ihh* expression, which normally promotes Runx2 expression in the perichondrium and the periosteum, which, in turn, is required for osteoblast differentiation.

The observation that the *Ihh* expression level was decreased in the epiphyseal cartilage of *Hand1-*overexpressing mice suggested that Hand1 could directly regulate *Ihh* expression. To address this possibility, we examined the effect of Hand1 on *Ihh* expression *in vitro* using ATDC5, a chondroprogenitor cell line, stably transfected with a *Hand1* expression vector. *Hand1* overexpression was confirmed in *Hand1*-transfected ATDC5 cells (Fig 4A). Expression of *Ihh* and *Runx2* in *Hand1*-overexpressing cells was significantly decreased (Fig 4A).

Runx2 directly binds and activates the *Ihh* promoter [2]. We have previously shown that Hand1 and Hand2 directly bind and inhibit Runx2 transactivation [15]. Because both Hand1 and Runx2 were expressed in the distal part of the immature chondrocytes of the cartilage primordium, and the expression pattern of Ihh was opposite to that of Hand1 (Fig 3A), it is possible that Hand1 negatively regulates *Ihh* expression through inhibited Runx2 activity. To address this possibility, we tested the effect of Hand1 on the transcriptional activity of the *Ihh* promoter. Overexpression of Hand1 or Hand2 in COS1 cells does not affect the expression level of Runx2 [15]. Hand1 alone did not affect *Ihh* promoter activity; however, it inhibited Runx2-dependent activation of the *Ihh* promoter (Fig 4B). Transfection of a Hand2 showed similar results to Hand1 (Fig 4B). To ensure this mechanism was unique to Hand proteins, we tested other tissue-specific bHLH proteins Id1, Tcf15, and Tcf21 in this assay. None of these proteins inhibited Runx2 transactivation of the *Ihh* promoter (Fig 4B). Runx2 induces the differentiation of perichondrial cells via the Ihh-Gli pathway [6]. These results suggest that Hand1 inhibits *Ihh* expression in the cartilage and consequently decreases *Runx2* expression in the perichondrium and the periosteum, where Runx2 is required for osteoblast differentiation (Fig 4C). Interestingly, misexpression of *Hand2* induces shortened and malformed limb in the absence of direct DNA binding [8]. Indeed, Hand1 and Hand2 do not require direct DNA binding to inhibit Runx2 transactivation function [15].

In summary, our results indicate that Hand1 is involved in proper osteogenesis of the bone collar via its control of Ihh production. Genetic overexpression of *Hand1* and *Hand2* in the osteochondral progenitors resulted in prenatal hypoplastic or aplastic ossification in the diaphyses. *Hand1* and *Hand2* overexpressing mice could provide unique animal models for understanding the molecular basis of limb development.

## Supporting Information

S1 Fig Defective ossification in Hand1 mutants. (DOCX)

S2 Fig Defective ossification in Hand2 mutants. (DOCX)

S3 Fig Chondrocyte morphology in femoral epiphyseal cartilage. (DOCX)

S1 Table Skeletal phenotypes observed in Hand1 mutant mice at P1. (DOCX)

S1 Text Supplemental Experimental Procedures. (DOCX)
