# Supplementary material for: The Transcription Factor Hand1 Is Involved In Runx2-Ihh-Regulated Endochondral Ossification
Source: PLoS One. 2016 Feb 26;11(2):e0150263. doi: 10.1371/journal.pone.0150263 (PMC4769249; doi:10.1371/journal.pone.0150263)
Supplement: S1 Fig — (DOCX) [file pone.0150263.s001.docx]

**
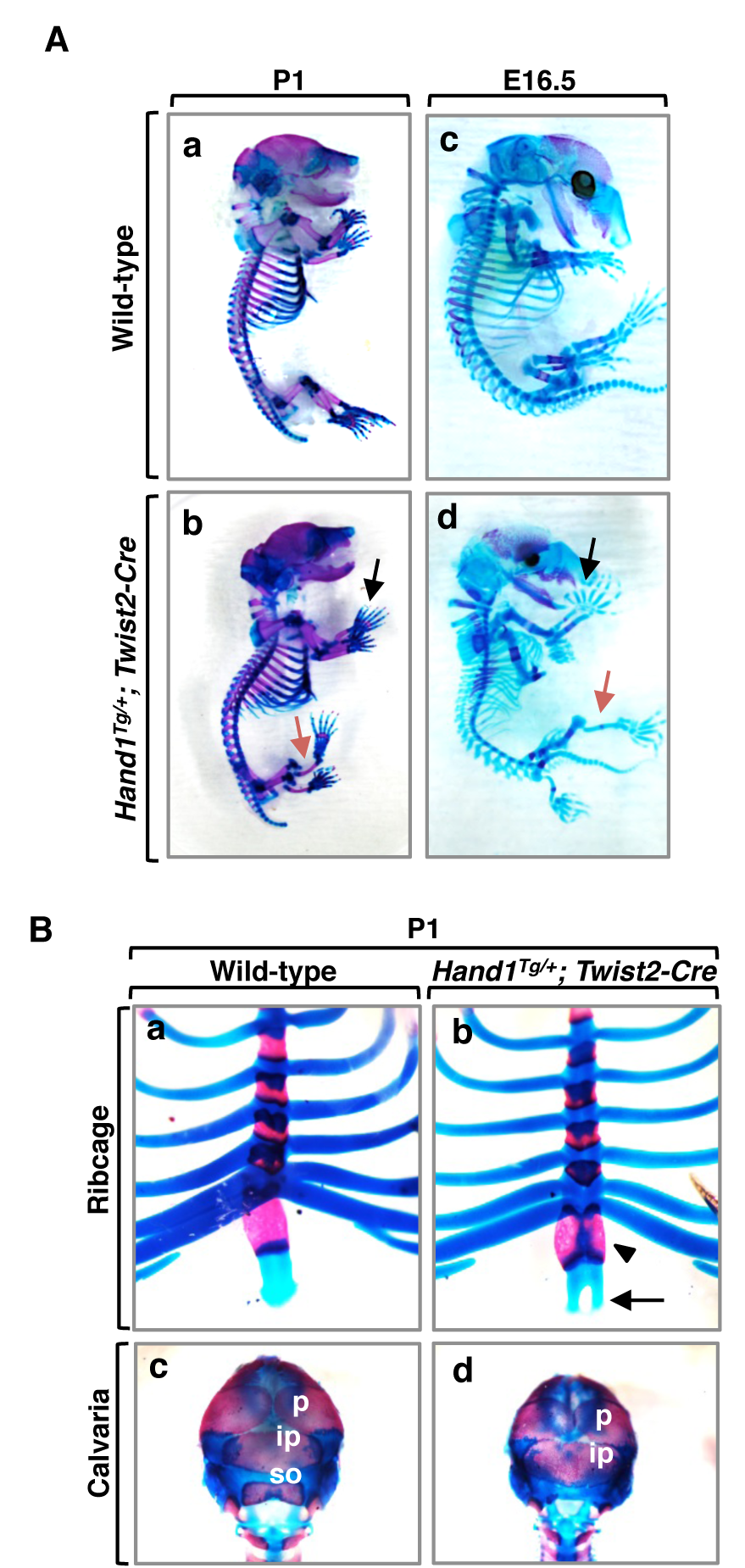
**

**S1 Fig. Defective ossification in *Hand1* mutants.**

(A) Skeletal phenotype of *Hand1*-overexpressing mice. Bone staining of wild-type (a,c) and *Hand1^Tg/+^; Twist2-Cre* mutants (b,d) at P1 (a,b) and E16.5 (c,d). Note the polydactyly in the mutant forelimb (black arrows in b,d) and congenital tibial aplasia in the mutant hindlimbs (red arrows in b,d). (B) Dissected ribcages (a,b) and calvaria (c,d) of wild-type (a,c) and *Hand1^Tg/+^; Twist2-Cre* mice (b,d) at P1. *Hand1* mutants show normal rib patterning (b), although the xiphoid process of *Hand1* mutants show hypoplastic ossification (arrowhead) and incomplete fusion (arrow) (b). The supraoccipital bone (so) is hypoplastic in *Hand1* mutants (b). p, parietal bone; ip, interparietal bone.
