# Supplementary material for: The Transcription Factor Hand1 Is Involved In Runx2-Ihh-Regulated Endochondral Ossification
Source: PLoS One. 2016 Feb 26;11(2):e0150263. doi: 10.1371/journal.pone.0150263 (PMC4769249; doi:10.1371/journal.pone.0150263)
Supplement: S2 Fig — (DOCX) [file pone.0150263.s002.docx]

**
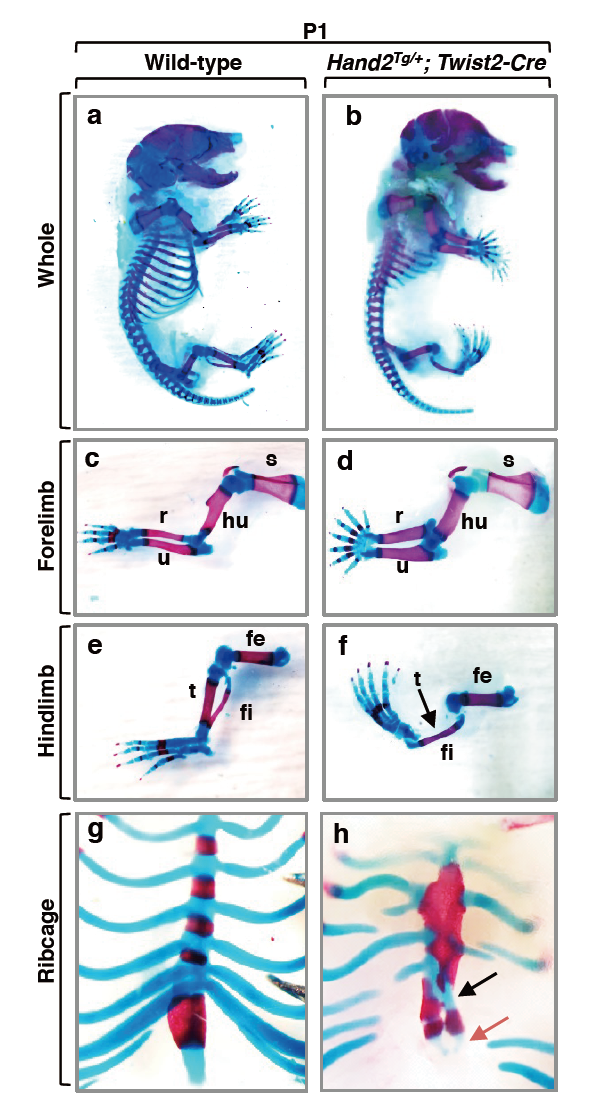
S2 Fig. Defective ossification in *Hand2* mutants.**

Skeletal phenotype of *Hand2*-overexpressing mice. Bone staining of wild-type (a,c,e,g) and *Hand2^Tg/+^; Twist2-Cre* mutants (b,d,f,h) at P1. *Hand2^Tg/+^; Twist2-Cre* mice show truncation of the zeugopod, as well as polydactyly in the forelimb (d). Note that the two cartilage epiphyses of the tibia (t) remain, whereas the middle calcified regions are aplastic (arrow in f). In the *Hand2^Tg/+^; Twist2-Cre* ribcage, abnormal rib patterning and formation, sternal ossification (black arrow), and failed formation of the xiphoid process (red arrow) are observed (h). r, radius; u, ulna; hu, humerus; s, scapula; fe, femur; t, tibia; fi, fibula.
