# Supplementary material for: The Transcription Factor Hand1 Is Involved In Runx2-Ihh-Regulated Endochondral Ossification
Source: PLoS One. 2016 Feb 26;11(2):e0150263. doi: 10.1371/journal.pone.0150263 (PMC4769249; doi:10.1371/journal.pone.0150263)
Supplement: S3 Fig — (DOCX) [file pone.0150263.s003.docx]

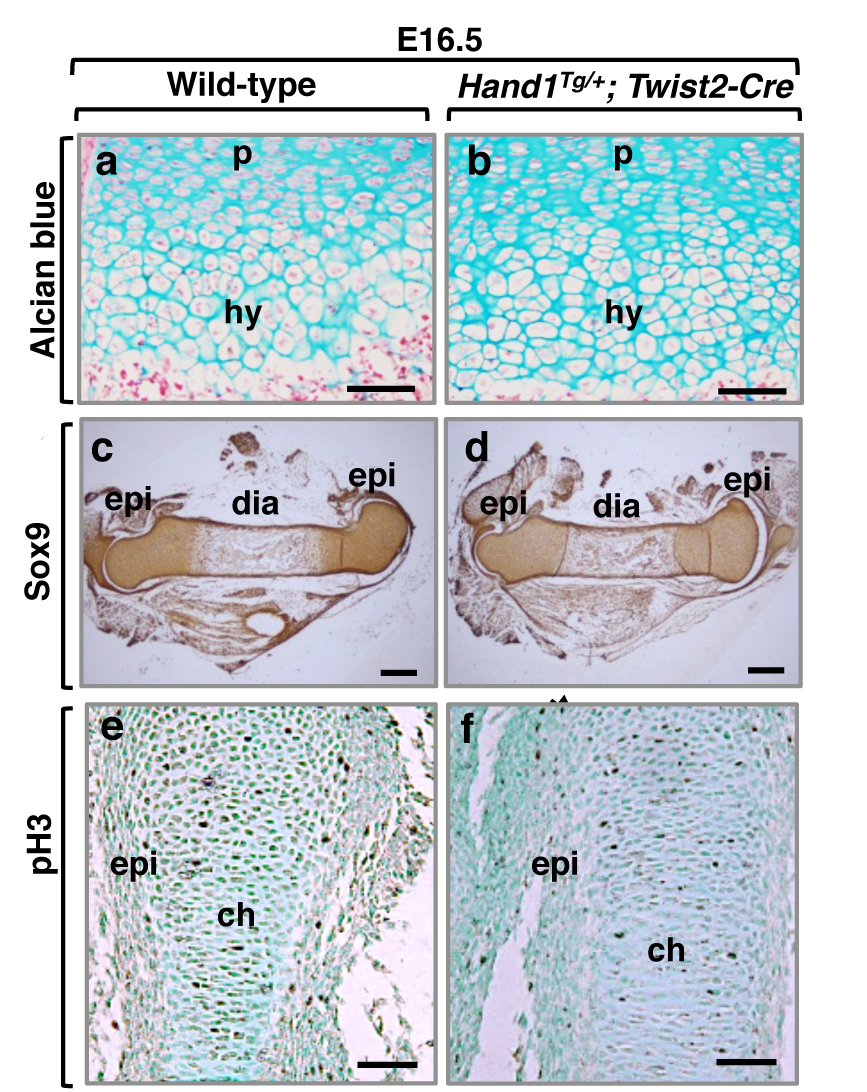
**S3 Fig. Chondrocyte morphology in femoral epiphyseal cartilage.**

(a,b) Alcian blue/Eosin Y staining of femoral epiphyseal cartilage isolated from wild-type (a) and *Hand1* mutants (b) at E16.5 (a,b). (c-f) Immunohistochemical analysis of Sox9 (c,d) and phospho-Histone H3 (e,f) in wild-type (c,e) and *Hand1* mutant femurs (d,f) at E16.5. There are no significant differences in detection of Sox9 or phospho-Histone H3 proteins between wild-type and *Hand1* mutant epiphyses. p, proliferating chondrocytes; hy, hypertrophic chondrocytes; epi, epiphysis; dia, diaphysis; ch, chondrocytes. Scale bars: 100 μm (a,b), 200 μm (c-f).
