# Supplementary material for: The Transcription Factor Hand1 Is Involved In Runx2-Ihh-Regulated Endochondral Ossification
Source: PLoS One. 2016 Feb 26;11(2):e0150263. doi: 10.1371/journal.pone.0150263 (PMC4769249; doi:10.1371/journal.pone.0150263)
Supplement: S1 Table — (DOCX) [file pone.0150263.s004.docx]

**S1 Table. Skeletal phenotypes observed in *Hand1* mutant mice at P1.**

|  |  | **Wild-type** | ***Hand1^Tg/+^;***  ***Twist2-Cre*** |
| --- | --- | --- | --- |
| **Forelimb** | Preaxial polydactyly | 0%  (0/13) | 100%  (13/13) |
|  | Mirror-image duplication of digits | 0%  (0/13) | 77%  (10/13) |
|  | Distal phalangeal duplication | 0%  (0/13) | 31%  (4/13) |
|  | Partial digits | 0%  (0/13) | 62%  (8/13) |
|  | Radial duplication | 0%  (0/13) | 69%  (9/13) |
|  | Hypoplastic ossification of radius | 0%  (0/13) | 100%  (13/13) |
|  | Hypoplastic ossification of ulna | 0%  (0/13) | 100%  (13/13) |
| **Hindlimb** | Polydactyly | 0%  (0/13) | 62%  (8/13) |
|  | Aplastic or hypoplastic ossification of tibia | 0%  (0/13) | 88.5%  (23/26) |
|  | Hypoplastic ossification of femur | 0%  (0/13) | 100%  (13/13) |
| **Xiphoid process** | Incomplete fusion | 14%  (1/7) | 54%  (7/13) |
|  | Hypoplastic ossification | 0%  (0/7) | 77%  (10/13) |
| **Supraoccipital bone** | Hypoplastic ossification | 0%  (0/13) | 85%  (11/13) |

Frequency (shown as percentage) and number (in brackets) of mice presenting skeletal patterning and ossification phenotypes at P1.
