# Supplementary material for: The Transcription Factor Hand1 Is Involved In Runx2-Ihh-Regulated Endochondral Ossification
Source: PLoS One. 2016 Feb 26;11(2):e0150263. doi: 10.1371/journal.pone.0150263 (PMC4769249; doi:10.1371/journal.pone.0150263)
Supplement: S1 Text — (DOCX) [file pone.0150263.s005.docx]

**Supplemental Experimental Procedures**

***Hand2* conditionally-overexpressing mice**

The transgene vector *CAG*-lox-*CAT*-lox-*Hand2* was constructed by inserting a *Hand2* cDNA into the *CAG-CAT-*(cDNA insert)-poly(A) cassette. The construct was injected into fertilized eggs to generate a permanent transgenic line, *CAG-CAT* *Hand2^Tg/+^* (Stock No. RBRC01366, RIKEN). For conditional activation of *Hand2*, *Twist2-Cre* males were crossed with *CAG-CAT* *Hand2^Tg/+^* females, producing *Hand2^Tg/+^; Twist2-Cre* mutants. Wt littermates were used as controls.
